# Raising and Releasing a Single Orphaned Harbour Seal Pup—A Case Report

**DOI:** 10.3390/ani16050851

**Published:** 2026-03-09

**Authors:** Guido Dehnhardt, Barbara Fölting, Yvonne Krüger

**Affiliations:** 1 Sensory & Cognitive Ecology Group, Department of Biosciences, University of Rostock, Albert-Einstein-Strasse 3, 18059 Rostock, Germany; yvonne.krueger@uni-rostock.de; 2 Marine Science Center, Am Yachthafen 3a, 18119 Rostock, Germany; barbarafoelting@hotmail.de

**Keywords:** harbour seal, orphaned seal pup, maternal separation, rehabilitation, stress mitigation, human care

## Abstract

Well-founded animal models of maternal separation show that early postnatal separation from the mother represents a massive stress factor for the offspring, with far-reaching negative effects on their physical and behavioural development. However, studies also show that the effects of maternal separation can be at least mitigated by appropriate physical attention. In this case report, we document the successful rehabilitation of a single orphaned male harbour seal pup by attempting to substitute natural maternal attention with intensive human attention. The pup underwent excellent physical development and displayed a close bond with its carers, characterised by intensive physical contact and suckling on a carer’s hand. However, during the final rehabilitation phase in a large outdoor enclosure, the pup increasingly reduced its affiliation to its carers. After its release, long-term observations over four years documented its hunting success, corresponding optimal physical development, and successful integration into existing wild seal groups. At the age of four, the now sub-adult seal displayed socio-sexual behaviour that is typical for this age and maintained a close bond with a wild female seal for months. The report shows that the rehabilitation of orphaned seals is possible by substituting maternal care with human care, resulting in neither a fixation on humans nor modifying species-specific behaviour. Thus, the pup’s chances of survival and potential reproduction in the wild were not jeopardised by this method of rehabilitation.

## Simple Summary

Many studies on animals, but also humans, show that early separation from their mothers causes considerable stress for pups and has negative physical and behavioural effects. At the same time, studies indicate that these consequences can at least be mitigated through appropriate physical attention. In this case report, we describe the successful rehabilitation of an orphaned male harbour seal pup, in which maternal care was replaced with intensive human care. The pup developed well physically and established a close relationship with its keepers, characterised by frequent physical contact and hand suckling. During the final rehabilitation phase in a large outdoor enclosure, the pup’s bond to its carers gradually diminished. Following its release, long-term observations over four years showed that the seal pup hunted successfully, developed well physically, and integrated well into existing wild seal groups. At the age of four, he showed the normal socio-sexual behaviour described for subadult male harbour seals and maintained a close bond with a wild female seal for several months. The report shows that the rehabilitation of orphaned harbour seal pups is possible by replacing maternal care with human care without the animal becoming attached to humans or altering species-specific behaviour, thus maintaining their chances of survival and eventually reproducing in the wild.

## 1. Introduction

**Figure 1:**
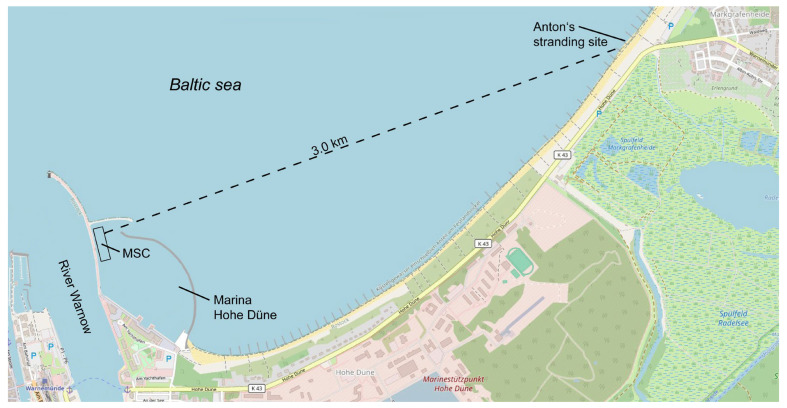
Modified map excerpt from “OpenStreetMap” showing the location of Rostock-Hohe Düne, the Marine Science Center (MSC) and the stranding site of Anton near the village of Markgrafenheide (Map data © OpenStreetMap contributors, available under Open Database License from www.openstreetmap.org/copyright).

On 30 June 2021, a male harbour seal (*Phoca vitulina*) pup (named Anton) was found on the beach of the village of Markgrafenheide, approximately 3 km from the Marine Science Center (MSC) in Rostock-Hohe Düne, Germany (Figure 1). It appeared to have lost contact with its mother, was very apathetic, and was classified as orphaned. Unlike in Lower Saxony and Schleswig-Holstein, Mecklenburg-Western Pomerania does not have an action plan or rehabilitation centre for such cases. The local veterinary authority therefore decided to first have the animal’s health examined at the Rostock Animal Clinic, which is experienced in treating the health issues of seals. Upon examination, the MSC was asked by the veterinary authority of the city of Rostock to attempt rehabilitation.

### 1.1. Orphaned Seal Pups Experience Early Postnatal Stress Due to Maternal Separation

Seal pups that have lost contact with their mother before becoming independent are naturally occurring, drastic cases of maternal separation. The effects of such separation have been the subject of research since the early 1950s, modelled as early ontogenetic stress. A brief overview of the complex findings of these studies will explain why we have provided Anton not only with food from the very beginning, but also with other elements of maternal care.

In both primates and rodents, maternal separation induces profound disruption of the hypothalamic–pituitary–adrenal axis (the HPA axis, also called the stress regulation axis) and causes complex effects, particularly on brain neurodevelopment and animal behaviour extending into adulthood (for review, see [1,2]). In a field study by Di Poi et al. [3], in which harbour seals were captured for research purposes, it was shown that the HPA axis’ reactivity with elevated serum cortisol levels is also present in harbour seal pups after a relatively short, complete maternal separation, whereas the presence of the mother during the capture process significantly attenuated these levels. This supports the assumption that the permanent maternal separation experienced by orphaned seal pups triggers a physiological stress response, not only due to the inevitable lack of food, but also due to the lack of maternal proximity and the associated care. There is therefore good reason to assume that the fundamental consequences of maternal separation described in animal models also apply to seals, even if, for example, species-specific predispositions and differences in early postnatal brain development may be present [1,4]. A recent study by Wilson et al. [5] investigated the stress factor to which the pups are exposed under various rehabilitation conditions by examining the effects of weight gain, social isolation, or free access to a water pool on the levels of various glucocorticoids. Cortisol, cortisone, prednisolone, and prednisone were measured in the animals’ urine to exclude the effects of handling during blood collection [6,7]. Interestingly, despite weight gain, the concentrations of the four glucocorticoids decreased in animals that also had free access to water. In contrast, just two of the four glucocorticoids, prednisolone and prednisone, decreased for pups without water access. This finding underscores the importance of this factor as a rehabilitation measure for developing seal pups, which under natural conditions can follow their mother into the water right after birth [8,9]. However, these results also suggest that the stress response can indeed also be influenced by external factors.

Considering that seal pups orphaned before weaning may spend days in the wild trying to re-establish their bond with their mother and, if admitted to a rehabilitation centre, usually spend at least several days in quarantine and thus continue to be socially isolated [5], it becomes clear that the physiological stress response associated with this prolonged isolation can have a massive impact on their development. Although the survival rate of rehabilitated seals in the first 5 months after release hardly differed from that of wild pups [10], these results do not allow for any conclusions to be drawn about whether and how these animals integrate into an existing population, which becomes important when they reach sexual maturity at the latest.

### 1.2. Long-Term Behavioural Effects of Maternal Separation: Insights from Other Mammals

To our knowledge, there are no studies regarding the stress effects of maternal separation on the long-term behavioural development of seal pups. However, the following comparative approach offers the possibility of approximating this assessment.

Early studies on rhesus monkeys (*Macaca mulatta*) [11,12,13,14,15] and pig-tailed macaques (*Macaca nemestrina*) [16] already showed that disruption of the intact bond between mother and offspring has a decisive impact on early development, but especially on the later personality of the young. In these studies, separations of mother and offspring were carried out, which, in terms of duration, are comparable to the maternal separation of orphaned seal pups, i.e., for at least several days, but also significantly longer. The impact on the behavioural development of the infants was quite consistent in anxiety disorders and depression-like apathy, a condition also known to exist in orphaned seal pups in advanced separation from their mothers [5]. However, the impact of early childhood isolation after sexual maturity on the maternal abilities of female monkeys was fatal [17]. Mothers who were raised without a mother showed little maternal care, instead ignoring or mistreating their offspring.

One might think that these results are primate-specific and can be explained by their relatively long-lasting mother–infant bond and highly complex social behaviour. However, in more recent research approaches, the effects of maternal separation are primarily investigated in classical laboratory animals such as mice and rats, by separating the offspring from their mothers early postnatally: for example, for 3 h per day. The relevant literature is extensive and methodologically diverse. The basic tenet here, however, is that maternal separation has profound effects on post-weaning behaviour, which, while varying depending on the species studied, sex, and research approach, generally results in complex anxiety-driven behaviour, comparable to those observed in the primates studied [4,18,19]. The comparability of the effects of maternal separation in such diverse mammals is also impressively demonstrated by the fact that, as in the rhesus monkeys of Harry Harlow and colleagues [17], female laboratory rats that experienced maternal separation early postnatally also showed deficits in the care of their offspring in adulthood [20]. In contrast, in adult male rats, the propensity for aggression is increased, which underscores the potentially sex-specific effect of maternal separation [21].

Regarding the behavioural development of rehabilitated harbour seals up to sexual maturity and beyond, these findings in other species certainly raise questions about the ability of these animals to integrate into natural populations. If maternal separation and prolonged isolation have comparable consequences for the maternal abilities of rehabilitated female seals, this would result in low survival rates for their offspring [22] and likely increase the rate of orphaned seal pups. Also worth considering are findings in rats showing that social defects induced by maternal separation not only occur in the animals directly exposed to this stress but are also passed on to their offspring for two subsequent generations [23].

With regard to the effect of maternal separation and the resulting stress response on brain development, the regulatory interaction of the HPA axis with the hippocampus is particularly important for species such as seals and other marine mammals with special spatial orientation requirements [24]. A chronic stress response with sustained cortisol release, as induced by maternal separation in seal pups [5], can impact the structural and functional development of the hippocampus, as well as other brain areas, thus resulting in impairments in spatial information processing and related cognitive abilities. For example, in laboratory mice that were separated from their mothers for only 24 h on the 9th day of life, a 20% reduction in neurons in the dentate gyrus of the hippocampus was observed [25]. Comparative tracking data from rehabilitated and wild seal pups are suggested to be interpretable in this direction [22]. They showed that the rehabilitated pups covered significantly greater distances daily during the tracking period [26,27], possibly due to orientation problems.

### 1.3. Implications for Seal Rehabilitation

The consideration of the consequences of maternal separation presented so far could provide a reason to forego any rehabilitation of orphaned seals, as was decided in Denmark. However, considering the avoidance of negative effects of maternal separation alone, there are also measures that give reasons for optimism. Of interest in this regard is the differentiated analysis of maternal separation effects in the rat model by Hofer [28,29]. He considers both maternal withdrawal as a stressor and the pup’s psychophysiological reactions to maternal separation as the sum of individual components, respectively. For example, a sufficient supply of milk for weight gain has a regulatory influence on the heart rate of isolated rat pups, while the provision of warmth affects behavioural activity, and pleasant tactile stimulation reduces aversive behavioural reactions due to spatial changes. The complex and profound effects of maternal separation on the offspring are considered to be a consequence of the *simultaneous* withdrawal of all maternal care components, such as food, warmth, and sensorimotor attention [28,29].

Breaking down the stressor ‘maternal separation’ into individual components suggests ways to mitigate its effectiveness by partially replacing maternal care. In laboratory rats, tactile stimulation alone during early postnatal isolation reduced physiological stress responses [30], and in mandarin voles (*Microtus mandarinus*), this sensorimotor attention reduced the pups’ otherwise prevalent anxiety and improved their cognitive performance and social behaviour [31]. Another important effect was that the young voles with tactile stimulation in isolation reached a higher body weight during the early development phase than control animals without tactile stimulation.

Applied to the rehabilitation of orphaned seal pups, these results suggest that it is possible to mitigate the consequences of maternal separation by replacing individual components of maternal care, but in any case, to contribute significantly to the normal development of the pups. In addition to pure food provision, it is therefore important to find ways to incorporate as many parameters of the natural mother–pup relationship into the rehabilitation process as possible. Wilson et al. [5] have shown the positive effect on the stress response for the pups having access to a pool; however, physical proximity and social interaction are of similar importance. Many seal rescue stations achieve the latter, for example, by socialising the animals after quarantine, which, however, means that the pups have already been in isolation for a very long time and have thus been exposed to the severe effects of maternal separation. Wilson and Alger [22], on the other hand, demonstrated how orphaned seal pups raised in pairs immediately after being found could function in some elements like a mother substitute for each other, practicing social behaviours and mutually mitigating the loss of their mother, to a certain extent. This effect has already been described in early studies on rhesus monkeys [13,32] and supports the comparability of early childhood social mechanisms across mammalian species.

### 1.4. Background and Objectives of Anton’s Rehabilitation, Release, and Long-Term Monitoring

The Marine Science Center is a research institute at the University of Rostock that primarily studies the sensory and cognitive mechanisms of marine mammal orientation; thus, in 2021, it was not equipped for the rehabilitation of marine mammals. However, since its staff had general expertise in all aspects of seal husbandry and experience in hand-rearing captive-born seals, the MSC gladly took on this task: naturally, with the primary goal of releasing the seal into the wild after successful rehabilitation, but also with the intention of incorporating our knowledge about the effects of maternal separation into the rearing process.

In this report, we document this successful rehabilitation and demonstrate that (i) despite intensive care and human attention, Anton did not develop a fixation on humans, and (ii) was able to hunt successfully after his release. After several months of absence following his release, Anton settled permanently in the outdoor area of the MSC, occasionally interrupted by several days, so that (iii) for the first time, the long-term development of a rehabilitated harbour seal and its integration into a wild seal population could be observed. It should be noted, however, that the rehabilitation of the seal pup was not designed as a scientific study, and here we are merely reporting on our experiences with the successful rehabilitation of a single orphaned harbour seal.

## 2. Material and Methods

### 2.1. Health Condition

Health examination at the Rostock Animal Clinic indicated that Anton weighed 9 kg, approximately 2 kg below the typical birth weight for harbour seals [33,34,35]. X-rays and blood tests showed no signs of infection, but the pup was extremely dehydrated and hypoglycaemic, confirming the apparently prolonged separation from its mother. Tachypnoea and tachycardia (heart rate > 220/min, see [36] for comparison) indicated a clear stress reaction. Since the animal was apparently healthy, except for the malnutrition, after initial treatment with a full electrolyte solution and prophylactic antibiotics, the animal was transported to the MSC.

### 2.2. Housing Conditions

For the rehabilitation of Anton, socialisation with neonate conspecifics was not possible. The MSC only keeps adult male harbour seals and eared seals, and we learned from our own experience that at least captive adult male seals are prone to infanticide in neonates. However, by separating the land portion of Anton’s outdoor enclosure with a net, visual and sporadic muzzle contact with our adult seals was possible. Aspects of maternal care were replaced by our own care, guided by the animal’s active role in the interaction [8].

**Figure 2:**
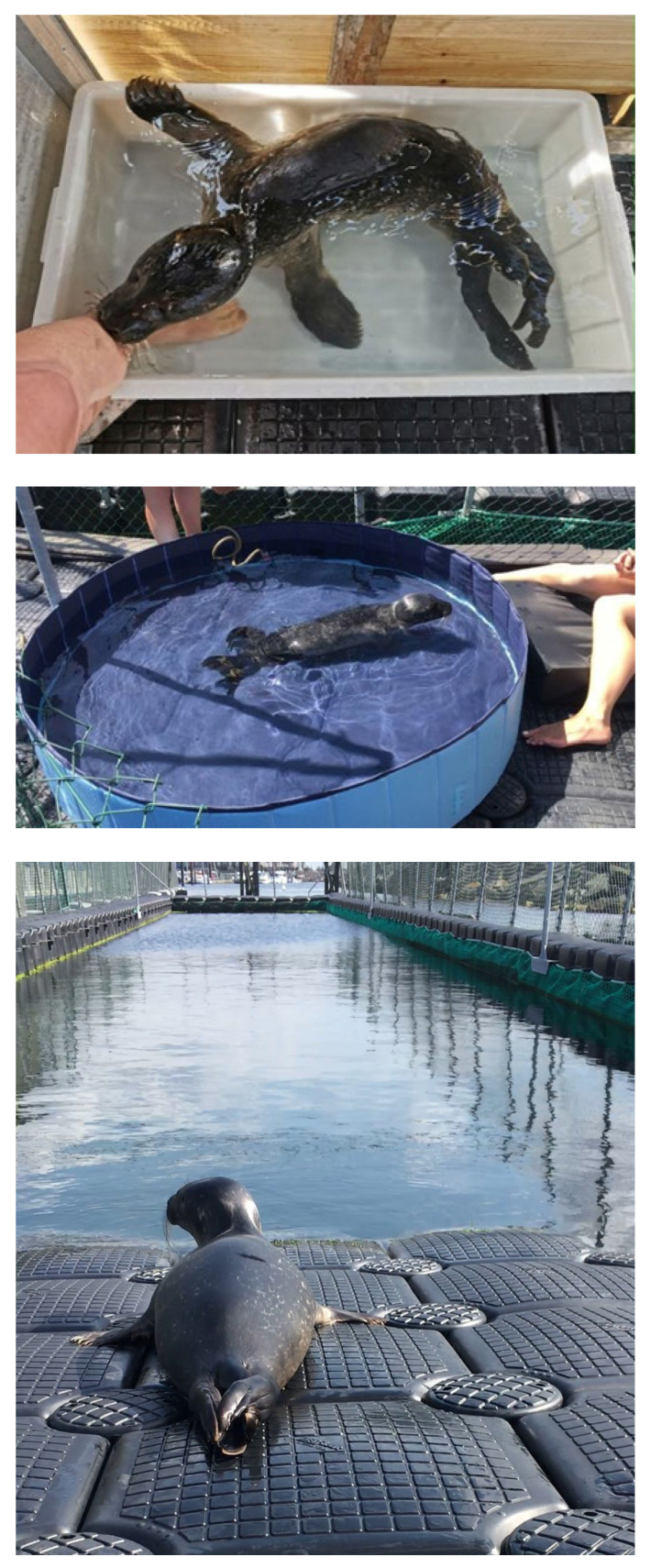
The three different water accesses during rehabilitation. (**top**): days 1–7, (**middle**): days 8–37, and (**bottom**): day 38 until release.

After arriving at the MSC on 30 June 2021, Anton was housed in one of our experimental huts (2.75 × 1.75 m), where he could be locked up at night for the first few days. This was necessary to protect him from American minks (*Neogale vison*), which populate the harbour pier in quite large numbers and even attack adult swans. For the first six days, we had to improvise and were only able to provide Anton with a tub of water (0.7 × 0.5 × 0.25 m, Figure 2 top) inside the hut, which was appropriate for his body length.

By the second day at the MSC, Anton was already very mobile and readily accepted the water tub via a small ramp made of sleeping mats. A 2 m^2^ rest area was provided, also made of sleeping mats, upon which several hot water bottles wrapped in towels were placed at night. In addition, an infrared heater was installed on the ceiling of the hut, which ensured a constant room temperature at night. The lying area was used by Anton especially overnight, so he was well-protected from hypothermia.

By the end of the first week, we had completed a 25 m^2^ outdoor enclosure made of wire mesh, in which we set up a circular pool with a diameter of 1.6 m and a water depth of 0.3 m, which was accessible via a ramp. From 8 July 2021 to 6 August 2021, Anton used this pool intensively on a daily basis (Figure 2 middle) and was particularly active in this area. However, he also spent a lot of time on land, mostly resting, from where he had a clear view of the large harbour basin and our adult seals. During periods of strong sunlight, he also entered the hut, which was accessible at all times.

On 7 August 2021, at an estimated age of 5 weeks, Anton was transferred to one of the MSC’s large side basins (35.0 × 5.5 m, water depth of 3–4 m), which was separated from the harbour basin and thus the Baltic Sea only by a coarse-mesh net (Figure 2 bottom). Due to the relatively large mesh size of our nets (6 cm), the MSC’s basins are always home to plenty of large shore crabs (*Carcinus maenas*), as well as smaller fish, such as juvenile garfish (*Belone belone belone*) and flounder (*Platichthys flesus*). Anton had access to a 4.0 × 5.0 m land area via a 2.5 m-long and 1.5 m-wide ramp that gently sloped into the water.

### 2.3. Nutrition

We based our diet on the experience gained from seal rescue stations. During the first nine days, Anton was fed 5 times a day (from 7:00 a.m. to 9:30 p.m., and on the first day also at 11:30 p.m.) every 3–4 h with pureed Baltic herring (*Clupea harengus membras*) via an intubation tube. At each feeding, Anton initially received 200–300 mL of fish mash, to which electrolytes (Saltadol, Aristo Pharma, Berlin, Germany), 5 mL of salmon oil (ReaVET, Schwalmtal, Germany), and once-per-day vitamins (0.5 Fish-Eater Tab 27373, Zoo Professionals, Natendorf, Germany) were added. Starting on the 10th day, the amount of fish mash was gradually increased to 350–400 mL per feeding, and at the second feeding of the day, Anton was fed 2 whole Baltic herring (totalling 100 g), which he swallowed without difficulty. A total of 5 mL of salmon oil was injected into the herring using a hypodermic needle. From day 13 to day 18, 300 g of Baltic herring were fed in two of the five feedings. Since gaining access to the round outdoor tank, Anton had been spending a lot of time in the water, so we threw individual herring into the tank. Anton picked up the herring lying on the bottom of the tank after a few minutes and swallowed them underwater. Starting on day 19, feedings were reduced to four times per day (8:00 a.m.–9:00 p.m.) and initially consisted exclusively of whole Baltic herring (approx. 400 g per feeding equivalent to 8 herring). This feeding schedule was maintained until release, but from 1 August 2021, the amount per feeding was increased to 700–800 g. In addition to herring, the total daily ration consistently included about one third sprats (*Sprattus sprattus*). Anton’s body weight was monitored by regular weighing. The weighing was performed by a carer standing on a calibrated scale, holding Anton close to her/his body, while another person read the weight from the scale.

## 3. Results

### 3.1. Physical Development

**Figure 3:**
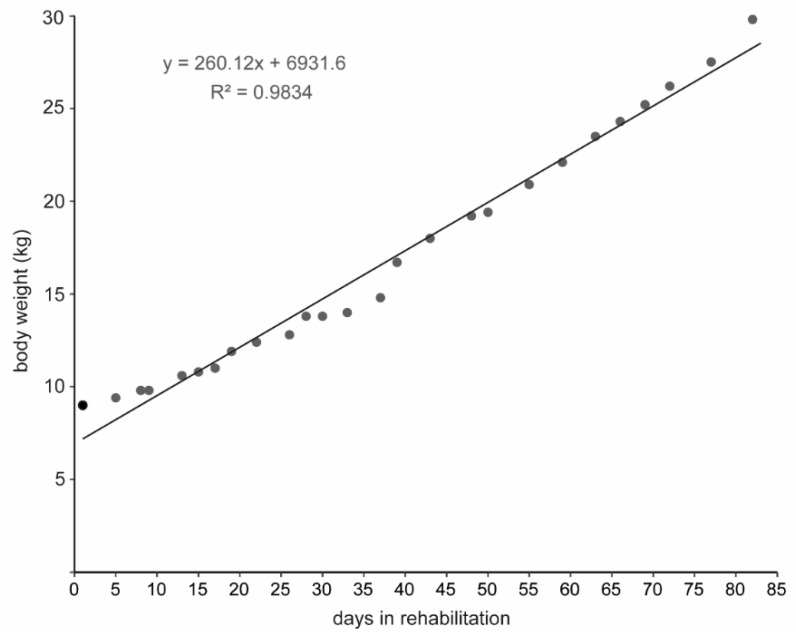
Anton’s weight gain during rehabilitation.

**Figure 4:**
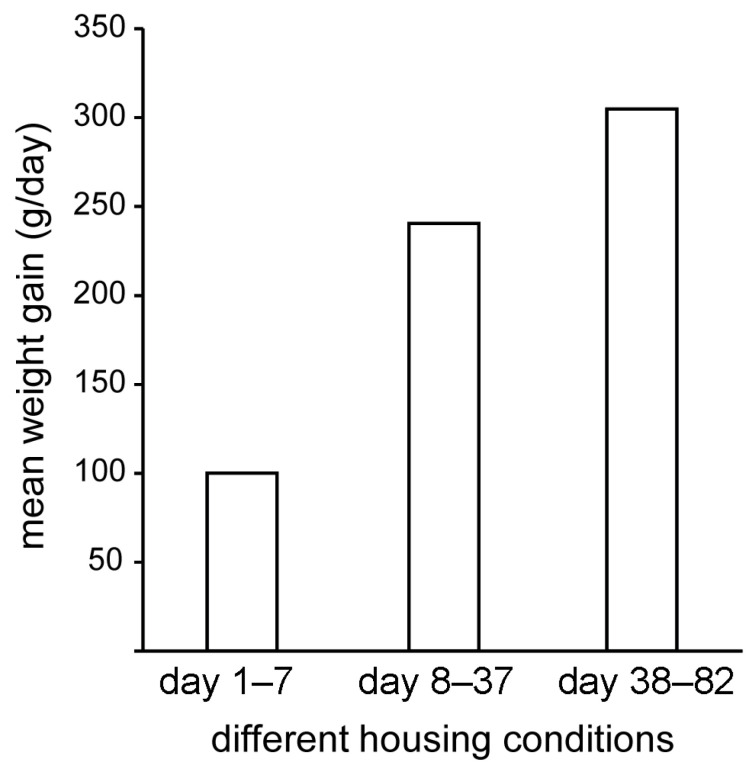
Mean weight gain during the three different housing conditions during rehabilitation: hut and water tub, days 1–7, outdoor with circular pool, days 8–37, large basin, days 38–82 and beyond until release.

Anton’s weight gain over the rehabilitation period is shown in Figure 3. However, the last weighing took place on day 82 after his arrival at the MSC, i.e., 17 days before his release. Weight gain was continuous throughout the entire period, stagnating for a few days (days 28–33), but never declined. In the first 8 days, in which he was only kept in the hut with access to the small pool (Figure 2 top), Anton gained a total of only 800 g, an average of 100 g per day (Figure 4).

In the second phase of rehabilitation, starting on day 8, in which Anton was kept predominantly outdoors with access to the larger water pool, the average daily weight gain increased to 240 g per day. During this time, however, the feeding regimen changed, with a gradual increase in the amount of fish mash and the introduction of whole fish (see above), which he received exclusively from the second half of the second phase of rehabilitation. With the transfer to the large open-water basin, the average daily weight gain increased to >304 g per day (Figure 4, day 38–82), even though we did not change the total amount of fish per day. However, Anton seemed to be consuming additional food from the abundant shore crabs.

### 3.2. Behavioural Development

From the second day after his arrival at the MSC, Anton was already very agile and began seeking physical contact with his carers. If the carers moved away from him after feedings, he would respond by vocalising “distress calls” and following them. Simply lying next to a person, even between feedings, was enough to allow Anton to calm down and stop vocalising. Of particular importance, however, was the opportunity to suckle on the body parts of a carer. As soon as he reached the person, Anton pressed his snout against the carer’s leg and began sucking with smacking noises (Figure 2 top). If the person withdrew, he immediately began vocalising again and tried to maintain contact with the person.

**Figure 5:**
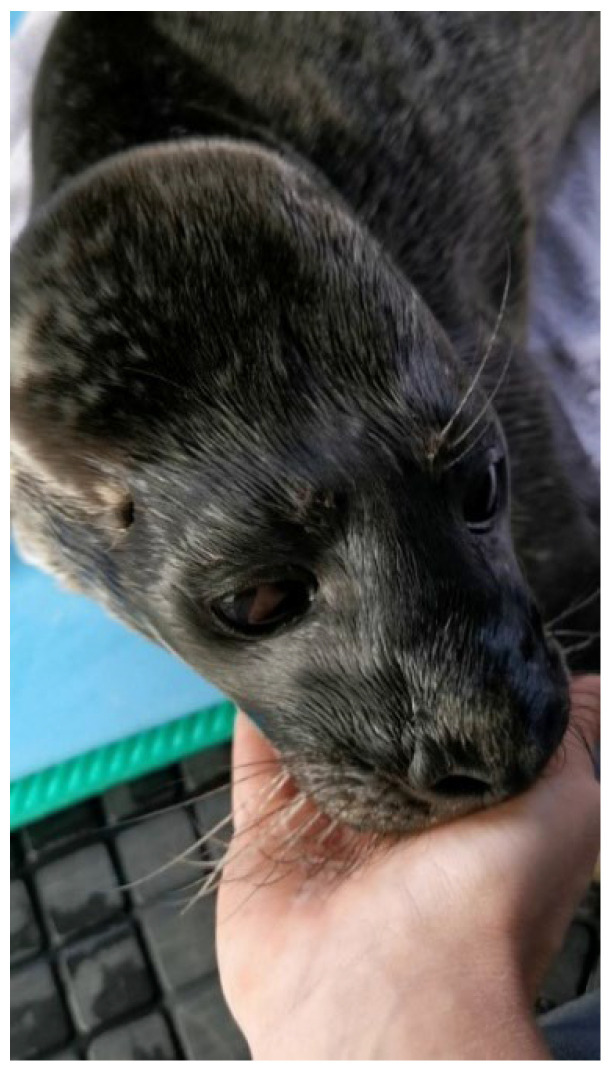
Anton suckling on the thenar of a carers hand.

It quickly became apparent that when Anton’s needs for physical contact and, above all, prolonged suckling were met, he became significantly calmer and hardly vocalised between feedings. Therefore, we systematically inserted a “suckling phase” after each feeding. Particularly after the last feeding late in the evening, Anton was allowed to suckle on the palm of his carer’s hand (hypothenar or thenar, see Figure 5), until he stopped suckling on his own, usually after 10–15 min. This way he became increasingly calm and sleepy and could then be left alone without becoming mobile or vocalising again. Monitoring for up to 3 h after the last feeding showed that Anton remained calm and, at least for that time, did not react aversively to being alone. The strong tendency to suckle remained relatively unchanged until day 30 of rehabilitation. Afterwards, Anton still sought the proximity of a carer, but nudged more with his snout than suckling.

This behavioural change prompted the initiation of the next phase of rehabilitation, which Anton was to spend in the large open water basin. This was realised on day 38 of rehabilitation and enabled Anton to develop his swimming and diving skills. After initial hesitation on the land area of this new enclosure, Anton quickly entered the water, but after several hours, he obviously had trouble getting back on dry land. Only when a carer lay down on the dry land did Anton dare to leave the water, seek out the carer’s proximity, and lie down relaxed next to the person. He needed this incentive from a carer to “haul out” for about a week, after which he also lay down on land on his own, but still sought the proximity of a carer as soon as the person entered the land part of his enclosure, which is why it was still easy to pick up Anton for weighing.

**Figure 6:**
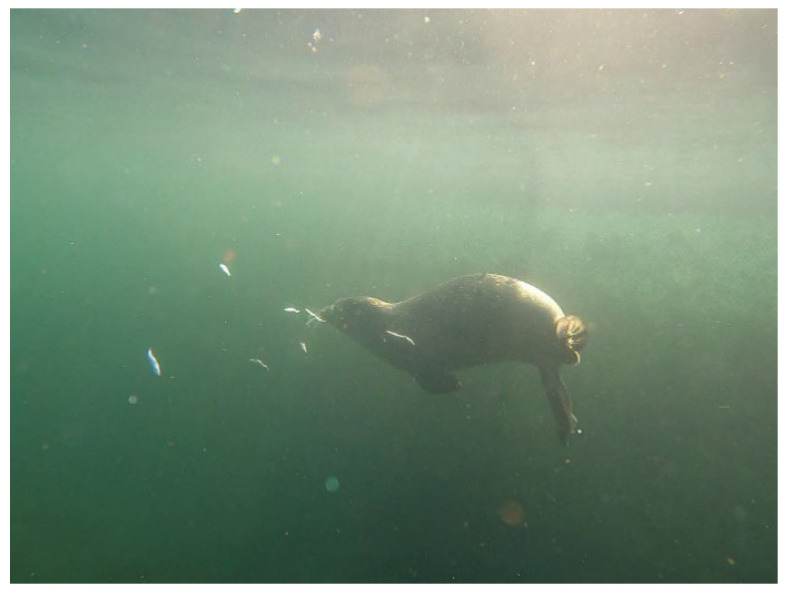
Anton playing hunting games with sprats.

During this time, Anton was fed only whole herring and sprats, sometimes by hand, but mostly by throwing the fish into the water. Underwater cameras showed that Anton sometimes created hunting games with the fish floating in the water, not simply eating them but pushing them through the water, then suddenly diving and “attacking” the fish from below and then eating it (Figure 6).

Starting on day 85 of rehabilitation, Anton’s behaviour toward carers changed. He no longer sought the proximity of a person and only hesitantly took fish from hand on land by stretching his neck out. Overall, his behaviour can be described as distant. Anton spent significantly more time diving than before and no longer interrupted dives when a person entered his enclosure. Weighing him would only have been possible if he had been caught, which is why this was not done, especially since he had already reached a weight of almost 30 kg at this point (Figure 3). This phase of alienation was not due to any external event and remained constant for another three weeks, so we decided to release him.

### 3.3. Release into the Wild and Further Development

**Figure 7:**
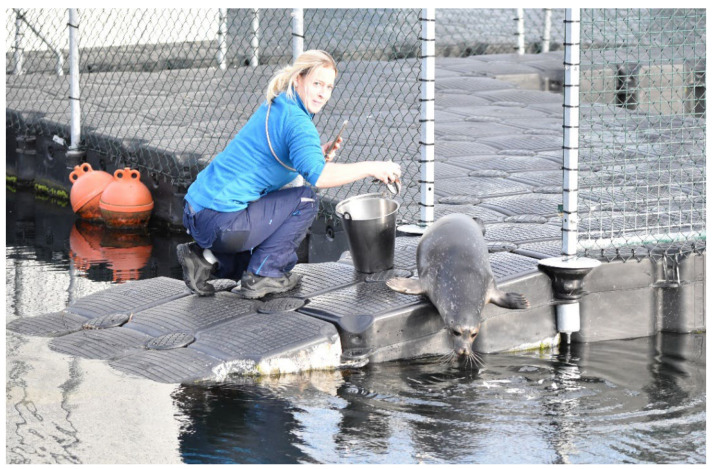
Anton leaves his enclosure, located between the east mole and the MSC’s Institute ship Lichtenberg, by following a handful of sprats that have been thrown into the marina.

On 6 October 2021, Anton was released after 98 days under human care. This timing was also favourable, because tourism along the coast had already decreased significantly, and less disturbance was expected in the harbour and surrounding area. Since Anton was found in the immediate vicinity of the MSC, it can be assumed that his birthplace is also nearby. His very low body weight of 9 kg on the day of admission supports this assumption, as it is unlikely that he was strong enough to follow his mother over long distances [37]. Nevertheless, the release site directly adjacent to the MSC represents a marine area about which Anton may have latently recorded and stored the environmental information necessary for spatial recognition and orientation. For the release, the outer door of Anton’s enclosure leading to the harbour basin was opened, allowing him to access the open water via a ramp. Anton was only briefly hesitant, but a few sprats thrown into the harbour basin quickly convinced him to follow them (Figure 7).

He did not attempt to return to his enclosure but immediately began exploring the entire area surrounding the MSC facility. On the second day after his release, he had already caught a flounder, successfully demonstrating his hunting abilities. Anton remained near the MSC for about eight weeks and was only occasionally absent for a day. However, he was also spotted in the river Warnow, for example, and thus made further excursions outside the harbour. He used the plastic pontoons installed around the MSC to stabilise the facility as resting areas. These pontoons are also often used by other wild harbour seals and grey seals (*Halichoerus grypus*). However, no other seals were present at the time of Anton’s release. In the weeks following his release, during which Anton remained near the MSC, no weight loss was observed. Due to the constant weight checks we conduct with the seals kept at the MSC, all staff members are trained to identify potential weight changes in our animals based on visual assessment and then verify them by weighing them. Anton also made no attempt to approach people, neither to beg MSC staff for food nor to approach tourists, for example, on land or on boats. Compared to other wild harbour seals that temporarily stay in the MSC area, the only noticeable difference in his behaviour was a shorter flight distance; in this respect, he was similar to the grey seals that rest at the MSC. After the end of November 2021, the exact date can no longer be reconstructed, Anton was not seen for five months, and there were no clues as to his whereabouts during this period.

**Figure 8:**
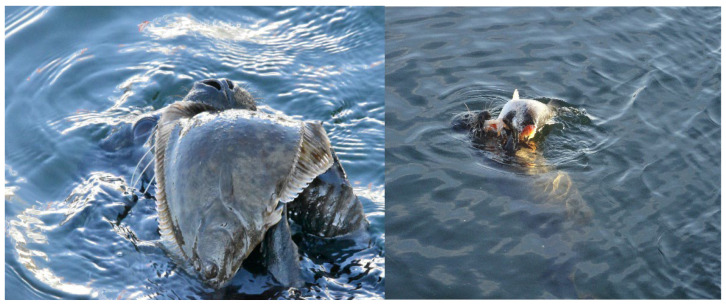
Anton at the age of about one year, successfully hunting flounder (**Left**) and sea trout (**Right**).

On 1 May 2022, after approximately five months of absence, Anton was again lying on one of the MSC’s outer pontoons. He was easily identifiable by his characteristic fur pattern, now significantly larger, and apparently in excellent condition. He continued to make no attempt whatsoever to approach MSC staff and even showed clear defensive reactions by growling and waving his front flippers when MSC staff had to pass Anton on the inside of the enclosure. In the period immediately after his return, Anton demonstrated his success in hunting, e.g., flounder and sea trout (*Salmo trutta trutta*) in the immediate vicinity of the MSC (Figure 8).

**Figure 9:**
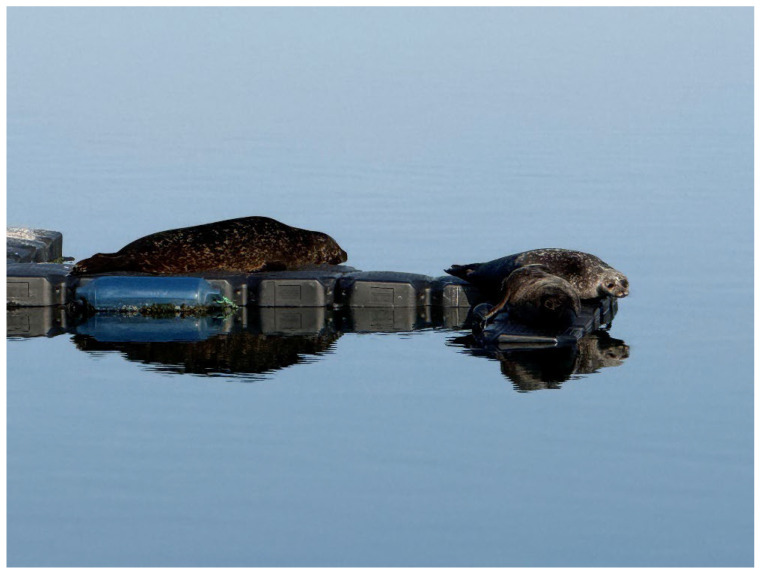
Anton (left seal) hauling out with two wild female harbour seals on pontoons of the outdoor facility near the bow of the Lichtenberg, the institute ship of the MSC, which is oriented south within the marina.

Since his return in May 2022, Anton has been a continuous resident at the MSC, with only short interruptions of a few days, allowing his physical and behavioural development to be monitored for more than three years. At the age of four (July 2025), he is already a good 150 cm long and is always in optimal nutritional condition. From late spring and summer 2024, i.e., at the age of just under 3 years, Anton began exhibiting courtship display behaviour or sexual play behaviour, as described by Venables and Venables [38], towards juvenile harbour seals by spinning on the spot underwater, creating chains of air bubbles, slapping his front flippers on the water surface, and making growling and barking noises. Since the winter of 2024/25, an increasing number of wild seals have been temporarily residing in the vicinity of the MSC, using the outdoor pontoons as haul-out sites. At times, there were up to eight seals, mostly juvenile, but also adult grey seals and harbour seals. Anton formed a close bond with two juvenile female harbour seals, interacting frequently with both of them in the water, and all exhibited typical juvenile courtship display behaviour [38]. The three also hauled out close to each other on the pontoons (Figure 9).

**Figure 10:**
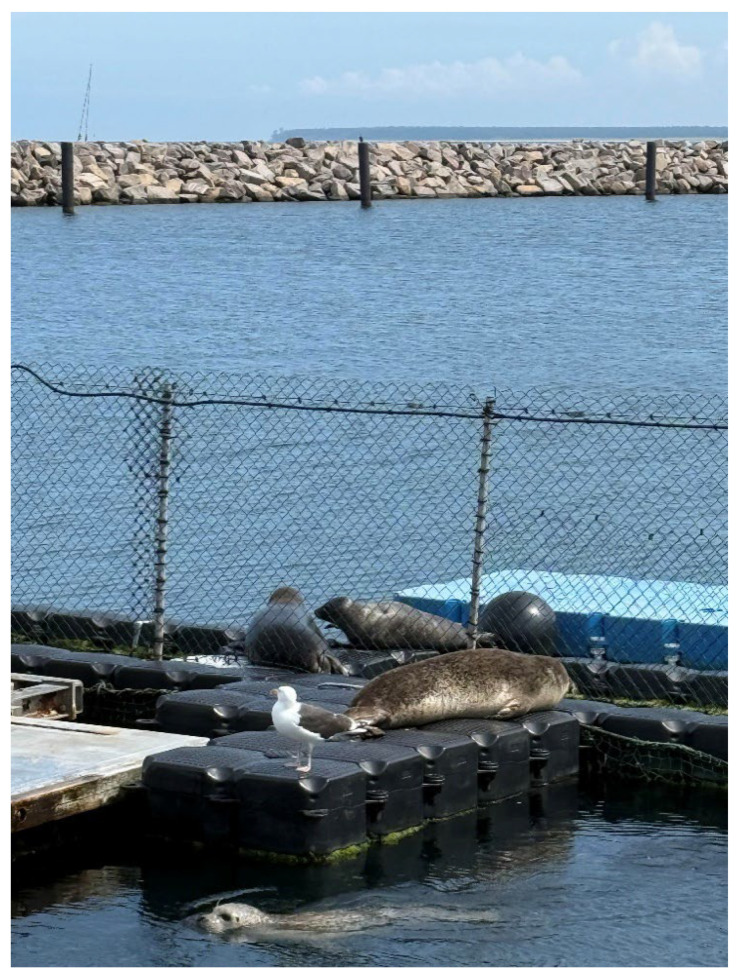
Anton (left) and the female Nugget (right) hauling out together on outdoor pontoons installed around the MSC facility. In the foreground, inside the enclosure, lies Nick, one of our research animals. Swimming in the water is Marco, who is 43 years old and the oldest seal at the MSC.

One of the females disappeared after a few weeks, while the other female, called Nugget, remained closely associated with Anton until October 2025 (the time of completion of this report). The proximity the two kept to each other, both in the water and especially during resting periods on land, is, at least in our experience, quite unusual for harbour seals and gives the impression of a strong pair bond. In the water, the behaviour patterns are difficult to attribute to individuals; when choosing resting spots, the initiative to stay together usually came from the female (Figure 10).

**Figure 11:**
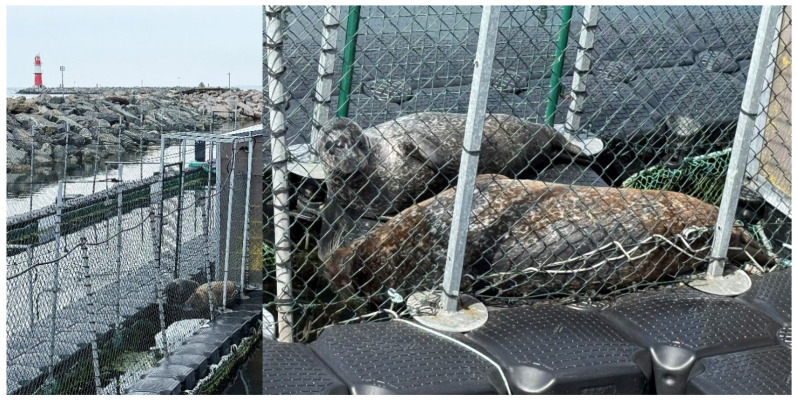
Anton and Nugget haul out together on a small platform located within a narrow channel between two enclosures near the stern of the Lichtenberg, the MSC’s institute ship. In the upper part of the left image, one can see the rocks of the east mole with the red lighthouse at the end.

Eventually, Nugget followed Anton to his favourite haul-out spot, a platform of just 1 m^2^ in a narrow channel (1 m wide) between two of the MSC basins located between the stern of the Lichtenberg, the MSC’s institute ship, and the east mole. The small platform was bordered underwater on both sides, down to the seabed by nets and above water by wire mesh (Figure 11).

From a seal’s perspective, this was an optimal haul-out site, as no approach was possible from three sides and the water could be reached as quickly as possible. However, in order to lie with their heads in the optimal escape direction, the two seals had to use an entrance area measuring only 0.5 m^2^. To allow the second seal to also access the haul-out platform, the seal that emerged first had to roll sideways on the narrow platform, otherwise it would have blocked the entrance from the water—an impressive feat of teamwork for two animals of a species that is often considered to be predominantly solitary.

## 4. Discussion

Although there are certainly species- and sex-specific differences regarding the consequences of maternal separation [2], studies spanning many decades indicate that the far-reaching detrimental effect of early postnatal separation of offspring from their mothers on individual development is a generally relevant principle in mammals. Supported by, in this regard, relevant stress hormone analyses [5], there is no reason to assume that similar effects cannot also be postulated for harbour seals. Wilson and Alger [22] first pointed out the possibility, but also the necessity, of successfully counteracting these negative effects in the rehabilitation of orphaned seal pups through, for example, housing them in pairs right after rescue. Our descriptions of Anton’s development over four years show that the rehabilitation of even a single orphaned seal pup is possible with intensive human care and attention, without jeopardising the survival chances of the released animal in the wild. On the contrary, with regard to his physical condition and all observable behavioural aspects, Anton shows no deviations from maternally raised harbour seals.

One crucial factor influencing Anton’s behaviour was presumably his access to a water pool from the beginning of his rehabilitation. Even exposure to the small water tub during the first week of rehabilitation, which just matched Anton’s body size, resulted in a significant increase in activity and the pup appeared more relaxed. This is consistent with the findings of Wilson et al. [22] that access to water decreases the glucocorticoid concentrations of harbour seal pups in rehabilitation. Regarding the physical contact that Anton actively sought during the first 30 days of rearing, the intensive suckling was of particular importance during this period. Especially after a feeding, i.e., when the pup was satiated, it had a calming effect that, if allowed to continue until the pup stopped, persisted for hours afterwards. This effect is comparable to the effect of tactile stimulation on mitigating maternal separation effects in altricial rodent pups [30,31]. The intensity with which Anton encouraged suckling is certainly part of the active role a seal pup plays in the mother–child bond [8]. With regard to the effects of maternal separation on the development of seal pups, not only the lack of maternal care plays a role, but also the suppression of the pup’s inherent behavioural components for actively maintaining the bond with the mother.

One reason often given for minimising any human contact with harbour seal pups undergoing rehabilitation is the concern that the animals will become habituated to humans or even become imprinted on them, with the phenomena of habituation and imprinting often not being distinguished. Scientific experiments, particularly on filial imprinting, such as the study by Shipley [39] of domestic guinea pigs (*Cavia porcellus*), are rare for mammals. The two existing studies on a juvenile California sea lion (*Zalophus californianus*, [40]) and a Steller sea lion (*Eumetopias jubatus* [41]) were not primarily designed to investigate the phenomenon of imprinting but rather inferred the presence of filial imprinting, based on various preference tests. Essential criteria for imprinting, such as the presence of a sensitive period, its irreversibility, and the exclusion of reinforcement mechanisms, could naturally not be considered or excluded. Since the rearing of sea lion pups by humans is comparable to cross-fostering experiments such as those conducted with sheep and goats [42], as well as with rodents [43], the persistence of the sea lions’ fixation on humans beyond sexual maturity, regardless of how it was initially formed, would have been particularly interesting. However, if filial imprinting is inherent in the behaviour of young otariids, this may not also apply to phocids [41]. Although Anton actively sought contact with his carers during rehabilitation, he showed no signs of filial imprinting or any other fixation on humans after release. He even responded to approaches with aggressive, dismissive behaviours such as “growling”, an “extended foreflipper” or a “foreflipper wave” [44]. Sexual imprinting, which only becomes apparent later, with the onset of sexual maturity, can also be largely ruled out, since Anton showed typical courtship behaviour of juvenile males at the age of four years [38], which already contains elements of the reproductive behaviour of adult seals [45,46,47,48,49]. Furthermore, he was successfully integrated into groups of wild seals in the area of the MSC exhibiting species-specific behaviours such as collective haul out.

It can be assumed that environmental factors such as the availability of prey caused Anton, similar to naturally weaned wild seal pups, to leave the MSC release site and travel for approximately five months. Tracking studies have shown that rehabilitated seal pups spend significantly more time in the water after release [10] and cover significantly greater distances both daily and over the entire tracking period than wild pups [26,27], which could explain Anton’s long absence. One reason for this difference is thought to be that wild pups receive behavioural training during their close bond with their mother [9,50]: for example, regarding hunting strategies or the selection and location of suitable haul-out sites. These complex orientation experiences are lacking in rehabilitated pups, who may compensate for this experience deficit during their significantly longer migrations without the role model function of their mother. In this context, the state of brain development during this early ontogenetic phase is also important as a basis for complex orientation skills. No data are available for harbour seals, but it has been shown that newborn Weddell seals (*Leptonychotes weddellii*), a similar precocial species, already possess over 70% of the brain mass of adult seals [51]. Although this is the highest value measured to date in mammals, the brain is not yet fully differentiated, and it is conceivable that early orientation experiences play a key role in subsequent neurogenesis. As studies on organisms as diverse as food-storing birds and ‘London taxi drivers’ [52,53] have shown, the hippocampus, in particular, a brain region crucial for spatial information processing, is characterised by plasticity, with the underlying neurogenesis induced by behavioural and cognitive demands as well as training through experience. A study by Zhuang et al. [54] found that captive-born spotted seals (*Phoca largha*) released at the age of two showed significant deviations from optimal habitat use compared to seals that had only undergone short-term rehabilitation at an age ≥ 1 year (age given only for three out of nine seals). Since it can be assumed that the captive-born spotted seals were raised by their mothers and maternal separation effects can thus be ruled out, the deficits in optimal habitat use after release could well be due to the limited challenges of spatial orientation during the early developmental phase in captivity and a resulting underdevelopment of brain areas that are crucial for spatial information processing. It remains speculation, but the time that wild harbour seal pups spend with their mother in their natural marine habitat could also be crucial for this neurological development process, so the longer stay in a greatly reduced captive environment could be a reason for the significantly longer migrations and the likely associated suboptimal habitat use of rehabilitated seals after release. In addition to these potentially experience-related effects on neuronal and cognitive development, it would be important to clarify whether maternal separation alone, as shown in other species [25], also has a detrimental effect on brain development, particularly on the hippocampus, in harbour seals and whether such a deficiency is reversible, for example, by extensive migration.

**Figure 12:**
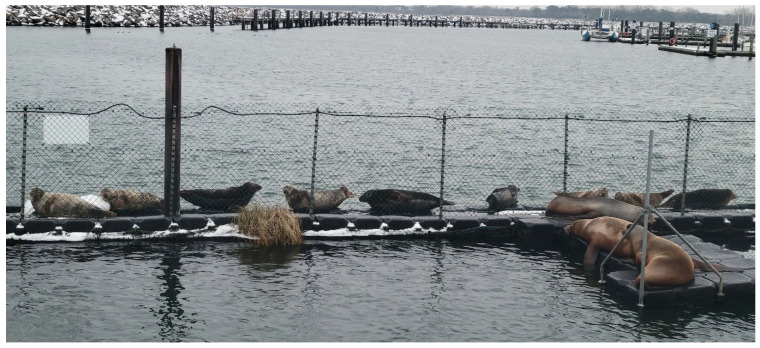
Nine wild seals hauling out on the pontoons surrounding the MSC enclosures, demonstrating the attractiveness of these resting places not only for Anton, but also for other animals of the wild seal population. In the foreground, inside the enclosure, lie the 3 eared seals of the MSC (photo by Jasmina Gebert, February 2026).

Harbour seals are generally characterised by a high degree of site fidelity [55,56], which can, however, vary regionally due to environmental factors such as the availability of suitable haul-out sites and food availability. In this respect, Anton’s site fidelity at the MSC is in line with this species-typical behaviour. Suitable, and above all undisturbed, haul-out sites are particularly rare for seals on the coast of Mecklenburg-Western Pomerania, so that the pontoons surrounding the MSC’s enclosure, from which boats within the marina must always keep their distance, offer an ideal alternative. This is also evident in the fact that an increasing number of wild grey seals and harbour seals are using these pontoons, at least temporarily, as haul-out sites (Figure 12). In addition, after his release and in the years following, Anton achieved sufficient hunting success in and around the harbour, so the site was characterised positively by two essential factors. The almost constant possible contact with conspecifics, especially females, may be a further factor in the attractiveness of the site.

In summary, the rehabilitation methods we used with Anton did not result in a fixation on humans, and, as long-term observation shows, did not result in any obvious deviations from the behaviour of a wild harbour seal. We are not aware of any studies that have followed the development of rehabilitated seals through sexual maturity, the critical phase of social integration. In Anton’s case, however, it has been demonstrated that, despite rehabilitation with close human contact, a male seal can achieve normal behavioural development close to adulthood. However, following the studies on maternal separation, it is necessary to explicitly demonstrate this for females up to the stage of mother–child relationships.

## Data Availability

The raw data supporting the conclusions of this article will be made available by the authors on request.

## References

[1] Lupien S.J., McEwen B.S., Gunnar M.R., Heim C. (2009). Effects of stress throughout the lifespan on the brain, behaviour and cognition. Nat. Rev. Neurosci..

[2] Zhang Y., Wang S., Hei M. (2024). Maternal separation as early-life stress: Mechanisms of neuropsychiatric disorders and inspiration for neonatal care. Brain Res. Bull..

[3] Di Poi C., Atkinson S., Hoover-Miller A., Blundell G. (2015). Maternal buffering of stress response in free-ranging Pacific harbour seal pups in Alaska. Mar. Mam. Sci..

[4] DeeAnn M., Reeder D.M., Kramer K.M. (2005). Stress in Free-Ranging Mammals: Integrating Physiology, Ecology, and Natural History. J. Mammal..

[5] Wilson S.C., Villanueva S., Jones K.A., Dmitrieva L., Smyth W. (2023). Urinary glucocorticoids in harbour seal (*Phoca vitulina*) pups during rehabilitation. Gen. Comp. Endocrinol..

[6] Trumble S.J., O’Neill D.O., Cornick L.A., Gulland F.M.D., Castellini M.A., Atkinson S. (2013). Endocrine changes in harbour seal (*Phoca vitulina*) pups undergoing rehabilitation. Zoo Biol..

[7] Dailey R.E., Smith K., Fontaine C., Jia Y., Avery J.P. (2020). Response of metabolic hormones and blood metabolites to realimentation in rehabilitated harbor seal (*Phoca vitulina*) pups. J. Comp. Physiol. B.

[8] Renouf D., Lawson J.W., Gaborko L. (1983). Attachment between harbour seal mothers and pups. J. Zool..

[9] Wilson S.C., Jones K.A. (2018). Behaviour of harbour seal (*Phoca vitulina vitulina*) mother-pup pairs in Irish Sea intertidal habitats. Biol. Environ..

[10] Lander M.E., Harvey J.T., Hanni K.D., Morgan L.E. (2002). Behavior, movements, and apparent survival of rehabilitated and free-ranging harbor seal pups. J. Wildl. Manag..

[11] Harlow H.F. (1958). The nature of love. Am. Psychol..

[12] Harlow H.F., Zimmermann R.R. (1959). Affectional responses in the infant monkey. Science.

[13] Harlow H.F., Kuenne Harlow M. (1962). Social Deprivation in Monkeys. Sci. Am..

[14] Hinde R.A., Spencer-Booth Y., Bruce M. (1966). Effects of 6-day maternal deprivation on rhesus monkey infants. Nature.

[15] Hinde R.A., Spencer-Booth Y. (1971). Effects of brief separation from mother on rhesus monkeys. Science.

[16] Kaufman I.C., Rosenblum L.A. (1967). The reaction to separation in infant monkeys; Anaclitic depression and conservation-withdrawal. Psychosom. Med..

[17] Seay B., Alexander B.K., Harlow H.F. (1964). Maternal behavior of socially deprived Rhesus monkeys. J. Abnorm. Psychol..

[18] Sánchez M.M., Ladd C.O., Plotsky P.M. (2001). Early adverse experience as a developmental risk factor for later psychopathology: Evidence from rodent and primate models. Dev. Psychopathol..

[19] Shin S., Lee S. (2023). The impact of environmental factors during maternal separation on the behaviors of adolescent C57BL/6 mice. Front. Mol. Neurosci..

[20] Lovic V., Gonzalez A., Fleming A.S. (2001). Maternally separated rats show deficits in maternal care in adulthood. Dev. Psychobiol..

[21] Veenema A.H., Blume A., Niederle D., Bauke Buwalda B., Neumann I.D. (2006). Effects of early life stress on adult male aggression and hypothalamic vasopressin and serotonin. Eur. J. Neurosci..

[22] Wilson S.C., Alger R. (2024). Mitigating the effects of maternal loss on harbour seal pups in captive care. Animals.

[23] Franklin T.B., Linder N., Russig H., Thöny B., Mansuy I.M. (2011). Influence of early stress on social abilities and serotonergic functions across generations in mice. PLoS ONE.

[24] Hanke F.D., Dehnhardt G. (2018). On route with harbor seals—How their senses contribute to orientation, navigation, and foraging. Neuroforum.

[25] Fabricius K., Wörtwein G., Pakkenberg B. (2008). The impact of maternal separation on adult mouse behaviour and on the total neuron number in the mouse hippocampus. Brain Struct. Funct..

[26] Gaydos J.K., Vilchis L., Lance M.M., Jeffries S.J., Thomas A., Greenwood V., Harner P., Ziccardi M.H. (2012). Postrelease movement of rehabilitated harbor seal (*Phoca vitulina richardii*) pups compared with cohort matched wild seal pups. Mar. Mam. Sci..

[27] Sangster S., Haulena M., Nordstrom C., Gaydos J.K. (2021). Interannual differences in post-release movements of rehabilitated harbor seal pups (*Phoca vitulina richardii*) in the Salish Sea. Mar. Mam. Sci..

[28] Hofer M.A. (2005). The psychobiology of early attachment. Clin. Neurosci. Res..

[29] Hofer M.A. (2006). Psychobiological roots of early attachment. Curr. Dir. Psychol. Sci..

[30] van Oers H.J., de Kloet E.R., Whelan T., Levine S. (1998). Maternal deprivation effect on the infant’s neural stress markers is reversed by tactile stimulation and feeding but not by suppressing corticosterone. J. Neurosci..

[31] Wei B., Tai F., Liu X., Ma L., Yang X., Jia R., Zhang X. (2013). Neonatal tactile stimulation alleviates the negative effects of neonatal isolation on novel object recognition, sociability and neuroendocrine levels in male adult mandarin voles (*Microtus mandarinus*). Physiol. Behav..

[32] Harlow H.F., Suomi S.J. (1971). Social Recovery by Isolation-Reared Monkeys. Proc. Natl. Acad. Sci. USA.

[33] Bowen W.D., Optedal O.T., Boness D.J. (1992). Mass and energy transfer during lactation in a small phocid, the harbor seal (*Phoca vitulina*). Physiol. Zool..

[34] Bowen W.D., Optedal O.T., Boness D.J., Iverson S.J. (1994). The effect of maternal age and other factors on birth mass in the harbour seal. Can. J. Zool..

[35] Cottrell P.E., Jeffries S., Beck B., Ross P.S. (2002). Growth and development in free-ranging harbor seal (*Phoca vitulina*) pups from southern British Columbia, Canada. Mar. Mam. Sci..

[36] Fonfara S., Casamian-Sorrosal D., Sundermeyer J., Rosenberger T. (2015). Variations in heart rate and rhythm of harbor seal pups during rehabilitation. Mar. Mam. Sci..

[37] Wilson S.C., Jones K.A. (2021). Body mass and behaviour of stranded harbour seal (*Phoca vitulina vitulina*) pups during the peak pupping season in Co. Down, north-east Ireland. Biol. Environ..

[38] Venables U.M., Venables L.S.V. (1959). Vernal coition of the seal *Phoca vitulina* in Shetland. Proc. Zool. Soc. Lond..

[39] Shipley W.U. (1963). The demonstration in the domestic Guinea pig of a process resembling classical imprinting. Anim. Behav..

[40] Schusterman R.J., Gisiner R., Hanggi E., Davis H., Balfour D. (1992). Imprinting and other aspects of pinniped-human interactions. The Inevitable Bond.

[41] Lynn B.L., Reichmuth C., Schusterman R.J., Gulland F.M.D. (2010). Filial Imprinting in a Steller Sea Lion (*Eumetopias jubatus*). Aquat. Mamm..

[42] Kendrick K.M., Hinton M.R., Atkins K., Haupt M.A., Skinner J.D. (1998). Mothers determine sexual preferences. Nature.

[43] Quadagno D.M., Banks E.M. (1970). The effect of reciprocal cross fostering on the behaviour of two species of rodents, Mus musculus and Baiomys taylori ater. Anim. Behav..

[44] Sullivan R.M. (1982). Agonistic Behavior and Dominance Relationships in the Harbor Seal, *Phoca vitulina*. J. Mammal..

[45] Venables U.M., Venables L.S.V. (1957). Mating behaviour of the seal *Phoca vitulina* in Shetland. Proc. Zool. Soc. Lond..

[46] Sullivan R.M. (1981). Aquatic Displays and Interactions in Harbor Seals, *Phoca vitulina*, with comments on mating systems. J. Mammal..

[47] Hanggi E.B., Schusterman R.J. (1994). Underwater acoustic displays and individual variation in male harbour seal, *Phoca vitulina*. Anim. Behav..

[48] Van Parijs S.M., Thompson P.M., Tollit D.J., Mackay A. (1997). Distribution and activity of male harbor seals during the mating season. Anim. Behav..

[49] Van Parijs S.M., Kovacs K.M. (2002). In air and underwater vocalizations of harbor seals in Eastern Canada. Can. J. Zool..

[50] Lawson J.W., Renouf D. (1987). Bonding and weaning in Harbor seals, *Phoca vitulina*. J. Mammal..

[51] Eisert R., Potter C.W., Oftedal O.T. (2013). Brain size in neonatal and adult Weddell seals: Costs and consequences of having a large brain. Mar. Mam. Sci..

[52] Sherry D.F., Hoshooley J.S. (2010). Seasonal hippocampal plasticity in food-storing birds. Philos. Trans. R. Soc. Lond. B.

[53] Maguire E.A., Gadian D.G., Johnsrude I.S., Good C.D., Ashburner J., Frackowiak R.S., Frith C.D. (2000). Navigation-related structural change in the hippocampi of taxi drivers. Proc. Natl. Acad. Sci. USA.

[54] Zhuang H., Tian J., Zhang Z., Wang Z., Zhao L., Lu Z. (2024). Challenges faced by spotted seals born in captivity and released into the wild. Glob. Ecol. Conserv..

[55] Dietz R., Teilmann J., Andersen S.M., Rigét F., Olsen M.T. (2013). Movements and site fidelity of harbour seals (*Phoca vitulina*) in Kattegat, Denmark, with implications for the epidemiology of the phocine distemper virus. ICES J. Mar. Sci..

[56] Cordes L.S., Thompson P.M. (2015). Mark-resight estimates of seasonal variation in harbor seal abundance and site fidelity. Popul. Ecol..

